# Assessment of the correlation between fluorescence-featured intraoral scanner, laser fluorescence and spectrophotometric analyses in caries-affected dentin: an in-vitro diagnostic accuracy study

**DOI:** 10.1007/s10103-025-04390-2

**Published:** 2025-03-14

**Authors:** Özlem Kanar, Bora Korkut, Dilek Tağtekin

**Affiliations:** Marmara University, Istanbul, Türkiye

**Keywords:** Caries removal, Color, Fluorescence, Scanner, Spectrophotometer

## Abstract

To investigate the relation between the caries scoring system of the fluorescence intraoral scanner (IOS) Trios 4 (3Shape, Denmark)a red laser light-induced fluorescence device DIAGNOdent Pen (Kavo, Germany), and the color parameters (L*a*b*) by a clinical contact type hybrid spectrophotometer Rayplicker (Borea, France) in the assessment of caries affected dentin tissue. Caries lesions were minimally invasively removal from 186 extracted molars. Teeth were scanned using Trios4, and the integrated software scored the cavity floor regarding the colors (yellow, initial caries; red, moderate-extensive caries) depending on the fluorescence features. Then the DIAGNOdent measurements were obtained from each cavity’s deep and discolored surface points following the previously obtained Trios4 colors. Thus, the Trios4 reading could be quantitatively assessed. Cross-polarization photographs were using the Rayplickerand L*a*b color parameters, and 3D Master (VITA) color mapping was obtained by using the RayPlicker’s software. Spearman’s Rho Correlation, Kappa, Mann Whitney-U, One-way Analysis-of-Variance, and Kruskal Wallis tests were used for the statistical analyses (*P* < 0.05). Sensitivity, specificity and AUC were calculated. Trios4 and DIAGNOdent were positively correlated (*r* = 0.733;*P* < 0.001). The L*parameter by Rayplicker and Trios4 was negatively correlated(*r*=-0.742;*P* < 0,001). The a*parameter by Rayplicker and Trios4 scoring positively correlated(*r* = 0.552;*P* < 0.001). No significant correlation was observed between b*parameter by Rayplicker and Trios4 (*r* = 0.023; *P* = 0.760). DIAGNOdent readings according toTrios4 scorings were significant (*P* < 0.001). The teeth without caries scored by the Trios4 corresponded to the median value of 29 in DIAGNOdent readings, and 88 for the teeth with caries (by Trios4 / yellow-red). The agreement between the Trios4 and DIAGNOdent was 52.2% for the specimens with no residual caries. The presence of caries scores by the Trios 4 corresponded to the DIAGNOdent readings of %100 for all the teeth evaluated. Regarding the RayPlicker assessments, 33% of the sound cavities corresponded to 2M3 color, and 73% corresponded to 5M3 color. Trios4 scorings presented 0.782 AUC, 56.30% sensitivity, and 100% specificity in DIAGNOdent reference. Trios4 scoring was considered coherent with the DIAGNOdent Pen. DIAGNOdent readings and the Level of L* and a* parameters in the dentin tissue might be considered interrelated. Fluorescence-featured scanner devices can be useful clinical tools to evaluate remaining dentin tissue during the caries removal procedure.

## Clinical trial number

Not applicable.

## Introduction

Non-invasive optical methods that do not involve ionizing radiation are gaining prominence for caries diagnosis. These non-invasive optical methods include visual examination, like ICDAS II criteria, where the caries stages are defined under adequate illumination [1]. After all, visual inspection is also an optical method based on the perception of the change in light transmittance of tooth hard tissues with increasing water content and decreasing mineral content under visible light. Accordingly, colorimetry and spectrophotometry systems in dentistry have been used to identify the carious tissue [2–7], in addition to the color studies examining bleaching systems [8, 9] and dental materials [10, 11]. The most current and valid method for color identification is the CIELAB system introduced by the International Commission on DDental Illumination in 1976 [12]. The CIE L*a*b* color system defines colors by three coordinates, which are “L*, a*, and b*”. The L* ”parameter” or “value refers to lightness, and the coordinates a* (red-green axis) and b* (yellow-blue axis) relate to the chromatic properties of the color. However, there is a lack in the literature regarding the assessment of carious dentin tissue using spectrophotometric analysis, which might be more useful in clinical dental practice.

There is no clear consensus on the endpoint of caries removal. However, some researchers agree that the main purpose of caries removal is to clean the dentin surface to the extent that the restorative material can be bonded [13, 14]. Schwendike et al. [14] recommended the selective removal of soft dentin in deep lesions (radiographically extending into pulpal 1/3 of the dentin). For shallow or moderate lesions, selective removal to firm dentin was recommended [14]. Additionally, caries activity markers like color (yellow/brown), or tactile examination (rough/or softness of the remaining dentin) also help the clinician determine the need for further intervention [15]. Studies have shown that preserving the remaining affected dentin, rather than the direct pulp capping, may reduce the need for endodontic treatment [16, 17]. Alleman et al. [13]. proposed the peripheral seal zone concept in the management of deep caries. This concept aims to provide caries-free area at the outer margins of the restoration while selective removal to soft dentin in central to preserve pulp exposure. If there is a risk of pulp exposure, it was suggested that central dentin stained by the caries detector dye, can be left for sealing if it is surrounded by 5 mm of sound dentin from the occlusal or 3 mm from the dentino-enamel junction.

Fluorescence-based systems like DIAGNOdent Pen (Kavo, Germany) also allow quantitative identification of caries lesions at earlier stages [18]. The system utilizes a principle based on the fluorescence change of dental tissue due to caries. Over the past twenty years, laser fluorescence recommended for the enamel caries diagnosis [18]. Studies revealed that the scores of the DIAGNOdent Pen device with the laser fluorescence feature had a positively agreement especially in situations where an increasing degree of enamel demineralization existed [19, 20]. According to the previous research, scores of 30 and above were determined for requiring restorative intervention for dentinal caries [18, 21, 22].

On the other hand, dentin staining has been reported to disturb the laser fluorescence values [23]. Neves et al. [24]. pointed out a significant negative correlation between the L parameter of the remaining dentin and and the DIAGNOdent Pen readings. In previous research, caries-infected and caries-affected dentine were described on digital images to determine caries removal efficiency, but no quantitative measurement such as lightness (L* parameter) was provided [25]. Another in-vitro study revealed a positive correlation between DIAGNOdent readings by and the L* value, but no correlation was found with the a* and b* parameters [6]. Yet, there are a limited number of studies in the literature with small sample sizes on the discrimination of dental caries using spectrophotometric characteristics and fluorescence spectrum [2, 6, 7].

Recently, another optical caries detection method was introduced which integrated in an intraoral scanner (IOS). Trios 4 (3Shape, Denmark) has a fluorescence that emits light at 415 nm [26]. In a previous study, the IOS-integrated fluorescence-based caries scoring system was found promising for the determination of caries stages [26]. However, the authors are unaware of any research on how this fluorescence-based system integrated into the IOS works after caries removal. The manufacturer has not provided information on whether the caries scoring system, developed with a formulation based on the fluorescence feature of the scanner, could be used after caries removal. Furthermore, it is unknown that the remaining caries defined by the Trios4 correspond to what color in the current color scales and the related DIAGNOdent readings after the caries removal. Another concern is the high probability of agreement between these non-invasive optical methods (Trios4, spectrophotometric color assessment, and DIAGNOdent readings), which might be useful information in clinical dental practice.

Based on this background, this in vitro study aimed to evaluate the agreement between a fluorescence-featured intraoral scanner Trios4, a red laser light-induced fluorescence device DIAGNOdent Pen, and a clinical contact-type hybrid spectrophotometer Rayplicker in analysis of caries affected dentin The null hypotheses were (a) no agreement was observed between the Trios4 scoring and DIAGNOdent readings, and (b) no relation was observed between Trios 4 scoring and the color parameters (L* a* b*) obtained by the Rayplicker.

## Methods

This in vitro study was approved by the local ethics committee with protocol number 09.2023.1727.

### Inclusion and exclusion criteria

**Fig. 1:**
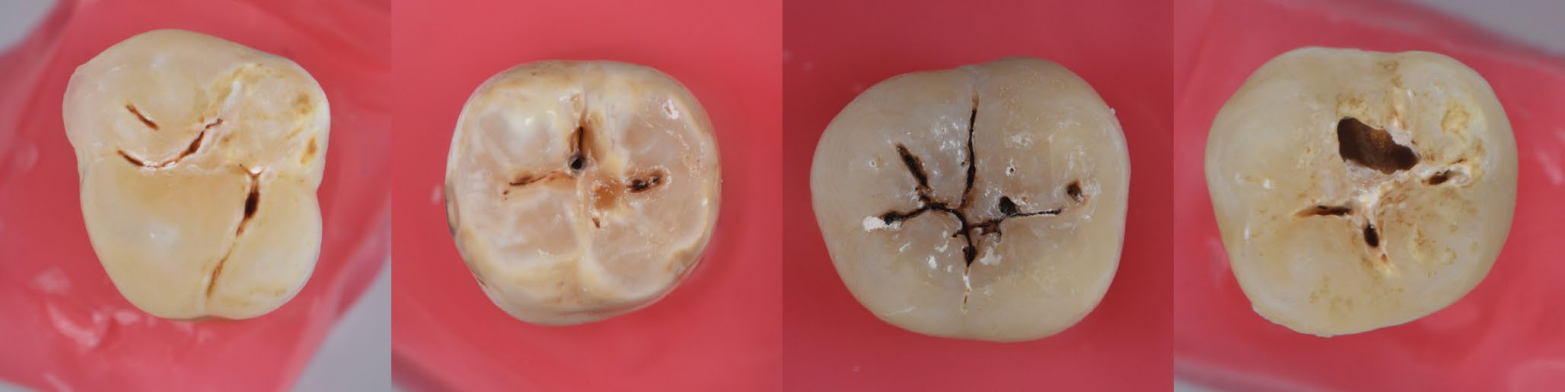
Photographs of the decayed teeth

Previously extracted human permanent molar teeth were included. The teeth were extracted for any reason in the last six months (caries or periodontal disease) and belonged to individuals aged between 18 and 40 years.The teeth were kept in 0.1% thymol solution until the beginning of the experiment. Before the experimental phase, the teeth were cleaned with pumice stone and prophylaxis paste. Then, each tooth was assessed visually and tactilely, then classified according to the International Caries Detection and Assessment System (ICDAS II) criteria [27–29]. One hundred and sixty-eight teeth occlusal caries lesions with (ICDAS 3, 4, and 5) were included in the study (Fig. 1). Primary teeth, cracked, worn, and sound or initial enamel lesions (ICDAS 0, 1, and 2), and severely damaged teeth (ICDAS 6) were excluded. Also, caries lesions on the buccal or proximal surface were excluded.

### Calibration

Before starting the experimental procedures, the operator trained by two senior researchers (BK and DT). BK has 20 years of experience in restorative dentistry and DT has 34 years of experience in restorative dentistry. The team has a large number of studies on caries diagnosis, caries removal, colour assessment and intraoral scanners. This training process included caries removal endpoint determination, intraoral scanner use and DIAGNOdent pen and Rayplicker evaluations. Training was carried out on 42 extracted molars. After caries removal, the operator confirmed the endpoint under the observation of senior investigators. After the training period, the operator achieved a high level of interobserver agreement (κ = 0.98 for BK and κ = 0.96 for DT) with the senior investigators for the endpoint after caries removal in all specimens. To calculate intra-observer agreement, all measurements were repeated 3 times by the operator. Intra-observer agreement for the operator was κ = 0.92 for DIAGNOdent measurements, κ = 0.96 for Trios 4 scanning and κ = 0.94 for Rayplicker assessment.

### Caries removal protocol

**Fig. 2:**
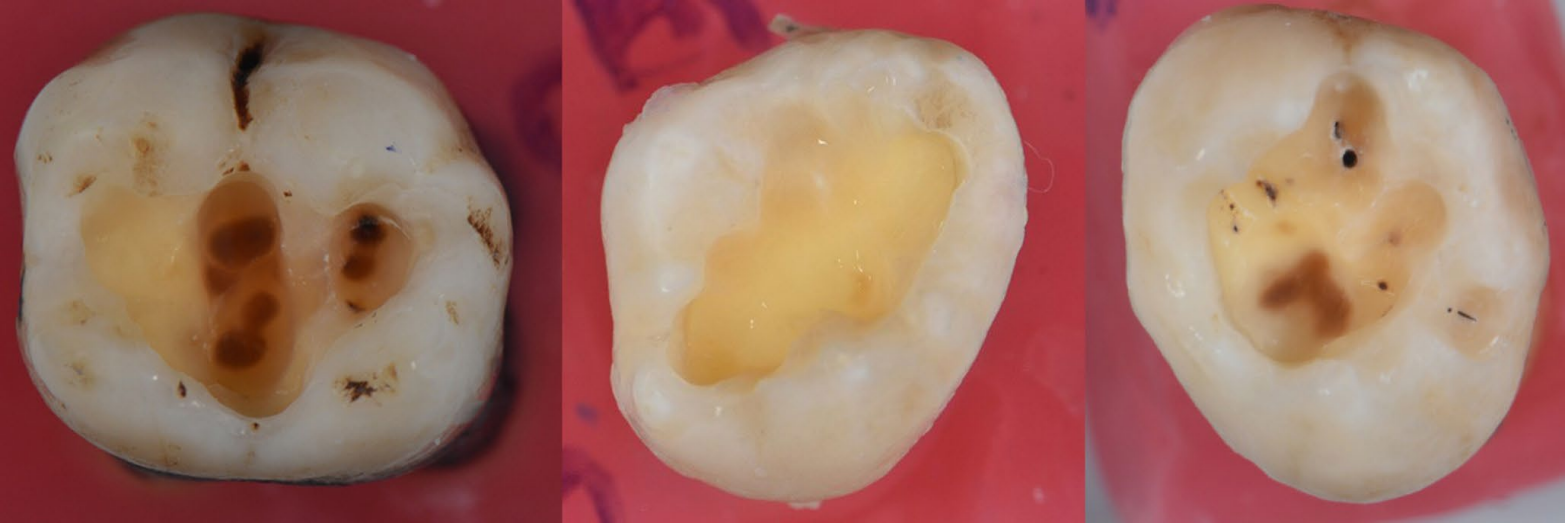
Photographs of the samples after caries removal

After the collection of extracted teeth, they undergo a caries removal phase. At first, carious enamel was removed by a round-shaped diamond bur in water cooling (#14 to #18) to reach the center of the dentinal lesion. Accordingly, a tungsten carbide bur (Frank Dental, Germany) was selected to remove the infected dentin. The most common method was adopted to represent the clinical routine: selective removal to soft dentin for deep lesions, and selective removal to firm dentin for shallow or moderate caries lesions [14]. The endpoint of caries removal is determined by visual and tactile examination. Accordingly, the prepared teeth were stored in 0.1% thymol solution until the scanning and spectrophotometric analysis were performed, to prevent color changes related the dehydration [30]. Photographs of the teeth after caries removal are available in Fig. 2.

### Caries assessment by Trios 4

**Fig. 3:**
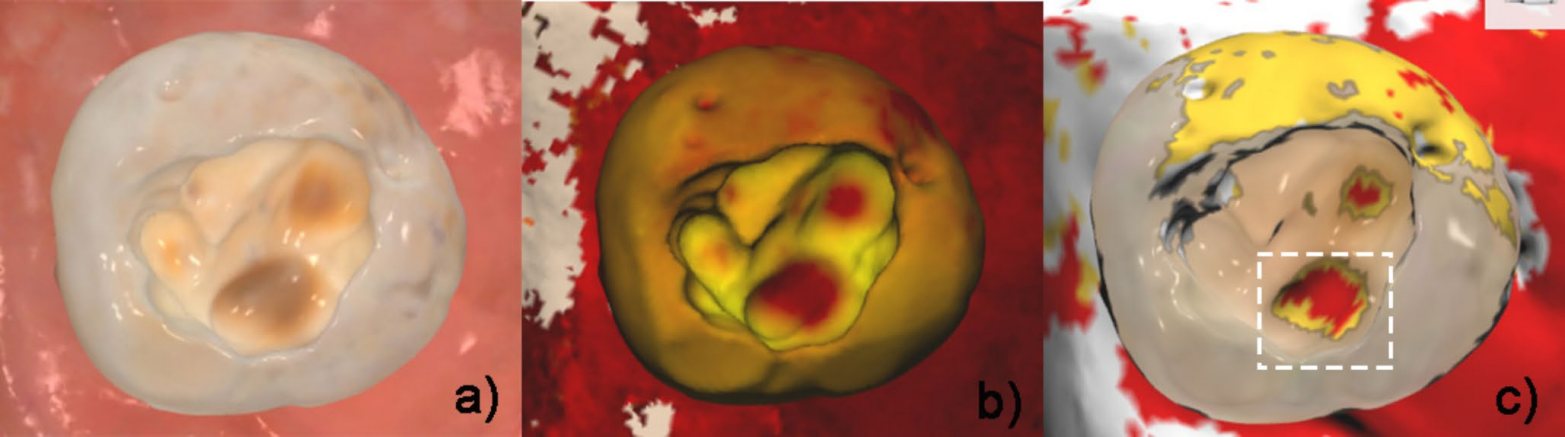
**a** to **c**; **a**: Trios4 screen after scanning, **b**: fluorescence screen of IOS (caries-aid tool) **c**: caries scoring software screen of IOS (Trios 4, 3Shape )

Each tooth was scanned with Trios 4 a fluorescence-based IOS, by the main investigator. During the scanning procedure, the teeth were kept humid. Scanning was performed as quickly as possible to avoid tooth dehydration and possible photobleaching [31]. All scanning procedures were performed in a dark box to simulate the light condition inside the mouth. The scanning time per tooth did not exceed 7 s. Subsequently, cavity floor fluorescence was visualized using the caries aid tool in the Trios4 software (Fig. 3). The software was allowed to ordinally score the caries lesion automatically by clicking on the “End Process” button. These scores were as follows: uncolored areas; intact, yellow; initial lesion, red; moderate/extensive lesions. The scan images were examined and the points to be measured were identified and marked, regarding the cavity floor regions scored by the IOS (Fig. 3c).

### Caries assessment by DIAGNOdent

**Fig. 4:**
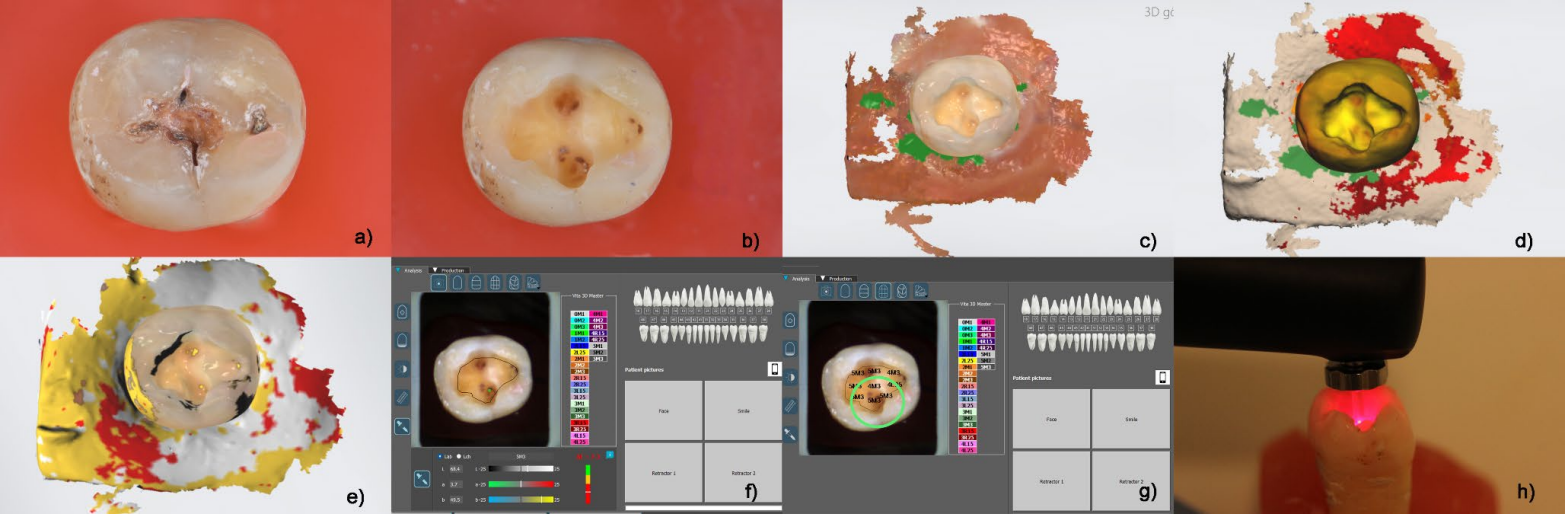
**a to h** Photographing the workflow **a**) Carious sample **b**) carious sample after caries removal **c**)Trios 4 IOS screen after scanning **d**) Fluorescence screen of Trios4: caries-aid tool **e**) Caries scoring system of Trios4 **f**) L*a*b* measurement of the scanner scoring point on Rayplicker (Borea Dental) software **g**) 3D master shade mapping by Rayplicker software **h**) LF reading from the same area (DIAGNOdent Pen, Kavo)

After the rehydration of the teeth, DIAGNOdent readings were performed at the marked sites on the cavity. The researcher made all measurements from the same point of the cavity and ensured standardization by marking the measurement area on the photograph. Figure 4 shows all the procedures and measurements that the extracted teeth underwent.

### Caries assessment by rayplicker spectrophotometric analysis

Accordingly, cavity floors were visualized with a clinical-type contact spectrophotometer (Rayplicker, Borea, Limoges, France). The measurements have been performed according to the manufacturer’s recommendations. Rayplicker’s own gray tip was used as a calibrator. The Rayplicker data were collected and transferred into the Rayplicker software, and L*, a* and b* parameters were measured from the previously marked regions, respectively. The corresponding color parameter of the corresponding region was then determined using the 3D Master mapping in the software. At all stages of the study, Trios 4 IOS recordings, DIAGNOdent readings and shade mapping performed while the teeth were rehydrated, and all the imaging were completed at the same time of day and in the same box to avoid errors due to light exposure.

### Statistical analysis

Data were analyzed with SPSS V23 (IBM, Armonk, NY, USA). The conformity of the data to normal distribution was examined by Kolmogorov-Smirnov and Shapiro-Wilk tests. Mann Whitney U test was used to compare the data that did not show normal distribution according to binary groups. One-way analysis of variance test was performed for the comparison of data conforming to normal distribution in the three groups, and multiple comparisons were analyzed with Tamhane’s T2 test. Kruskal Wallis H test was used to compare the data that did not conform to normal distribution in three groups, and multiple comparisons were performed by Dunn’s test. Spearman’s rho correlation coefficient was used to examine the relation between continuous parameters that did not conform to normal distribution. Kappa test statistic was used to examine the agreement between Trios4 caries status and DIAGNOdent caries status. Sensitivity, specificity, accuracy, Area Under Curve (AUC), positive predictive value (PPV) and negative predictive value were calculated for Trios 4 according to DIAGNOdent. Analysis results were presented as frequency (percentage) for categorical variables, mean ± standard deviation, and median (minimum-maximum) for quantitative variables. The significance level was set at *P* < 0.050.

The present study was completed with 186 dentin surface evaluations with 95% confidence (1-α), H1 = 0.733, and the power of the test (1-β) was obtained as 100% as a result of post-hoc power analysis.

## Results

There was a statistically significant positive correlation between IOS scoring and DIAGNOdent readings (*r* = 0.733; *P* < 0.001, Table 1). DIAGNOdent readings also differed by IOS score (*P* < 0.001) (Table 2). The median DIAGNOdent reading was 29 for specimens determined by the IOS as “sound” and 88 for specimens determined by the Trios4 as “carious”.

**Table 1:** The relation between Trios4 scoring and LF readings

	LF reading	
	r*	*p*
IOS score	0.733	**< 0.001**

* Spearman’s Rho correlation coefficient

**Table 2:** DIAGNOdent Readins according to Trios4 scoring

	LF reading			
	Mean ± s.d	Median (min-max)	Test statistic	*p*
IOS Score				
Sound	36.00 ± 21	29 (4–99)	7.573.000	**< 0.001**
Caries	81.00 ± 20	88 (38–99)		

*Mann Whitney U test

There is a highly significant negative correlation between L-parameter and DIAGNOdent readings (*r*=-0.780; *P* < 0.001). A positive and highly statistically significant correlation was observed between the “a” parameter and DIAGNOdent readings (*r* = 0.601; *P* < 0.001). There was a positive but weak significant correlation between b parameter and DIAGNOdent readings (*r* = 0.157; *P* = 0.033) (Table 3).

**Table 3:** The relation between L a B values and diagnodent readings

	DIAGNOdent reading	
	r*	*p*
L	-0.780	**< 0.001**
a	0.601	**< 0.001**
b	0.157	**0.033**

* Spearman’s Rho correlation coefficient

The median “L” parameters showed a significant difference regarding the Trios4 scoring (*P* < 0.001). According to the Trios4 scoring, the median L parameter is 72.1 for sound, 48.55 for initial lesions, and 38 for moderate/extensive lesions. The median L parameter differs between sound and initial Trios4 lesion scoring and between sound and moderate/extensive Trios4 scorings (Table 4). However, the median L parameter does not differ between those with initial and moderate/extensive Trios4 scoring.

**Table 4:** L, a and B parameters according to Trios4 scoring

		IOS score			Test stat.	*p*	r***;*p*
		Sound	initial	moderate/extensive			
L	Mean ± s.d	70.31 ± 11.31	49.37 ± 10.54	40.15 ± 11.09	102.096**	**< 0.001**	-0.742
	Median	72.1	48.55	38			**<0.001**
	(min. - max.)	(32–84)^a^	(34–75.5)^b^	(18–68.5)^b^			
a	Mean ± s. d	4.76 ± 7.56^a^	12.07 ± 3.7^b^	14.25 ± 4.26^b^	51.969*	**< 0.001**	0.552
	Median	4	12	12.9			**< 0.001**
	(min. - max.)	(-9.5–24)	(5.6–22)	(7.1–23)			
b	Mean ± s. d	26.33 ± 9.95	32.35 ± 8.63	25.3 ± 9.85	9.879**	**0.007**	0.023

a: b There is no difference between those with the same letter.*One-way Analysis of Variance.**Kruskall Wallis Test.***Spearman’s Rho Correlation Coefficient

The mean a* parameter showed a significant difference according to Trios4 scoring (*P* < 0.001). The mean a* parameter was 4.76 in samples scored as “sound”, 12.07 in those scored as “initial”, and 14.25 in those scored as “moderate/extensive”. The mean a* parameter differed between those with “sound” and “initial“ Trios4 scores and between “sound” and “moderate/extensive” Trios4 scores. However, mean a parameter did not differ between initial and moderate/extensive Trios4 scores (Table 4).

The median b* parameter differed according to Trios4 scoring (*P* = 0.007). The median b* parameter was 27 in those with sound, 34 in those with initial, and 26.7 in those with moderate/extensive lesions. The median b* parameter differed in those with sound and initial Trios4 scoring and in those with initial and moderate/extensive Trios4 scoring. However, the median b parameter was not significantly different in those with sound and moderate/extensive Trios4 scoring.

A statistically significant, highly negative correlation was observed between L parameter and IOS scoring (*r*=-0.742; *P* < 0.001). There is a statistically significant, highly positive correlation between a parameter and Trios4 scoring (*r* = 0.552; *P* < 0.001). However, no significant correlation was observed between the b parameter and Trios4 scoring (*r* = 0.023; *P* = 0.760).

When the shade matches of carious and non-carious samples were examined according to DIAGNOdent readings, it was found that 55% of the samples read as non-carious corresponded to shade 2M3, followed by shade 4R25 at 20%. It was found that 57.9% of the samples identified as carious by DIAGNOdent readings corresponded to shade 5M3. This was followed by the 5M1 shade at a rate of 13.5%.

When the shade matches of carious and non-carious samples were examined according to DIAGNOdent readings, it was found that 55% of the samples read as non-carious by DIAGNOdent corresponded to shade 2M3, followed by shade 4R25 at 20%. 57.9% of the samples identified as carious by DIAGNOdent readings corresponded to shade 5M3 followed by the 5M1 shade at a rate of 13.5% (Table 5).

**Table 5:** 3D master scale distribution according to diagnodent readings

Vita Master scale	DIAGNOdent reading	
	≤ 30	> 30
	*n* (%)	*n* (%)
2L25	0 (0)	1 (0.8)
2M2	1 (1.7)	0 (0)
2M3	33 (55)	3 (2.4)
3L25	2 (3.3)	5 (4)
3M3	1 (1.7)	8 (6.3)
3R25	3 (5)	2 (1.6)
4M3	0 (0)	5 (4)
4R15	1 (1.7)	0 (0)
4R25	12 (20)	9 (7.1)
5M1	4 (6.7)	17 (13.5)
5M2	0 (0)	2 (1.6)
5M3	3 (5)	74 (57.9)

A statistically significant, moderate agreement was observed between Trios4 scoring and DIAGNOdent readings (κ = 0.454; *p* < 0.001). The rate of specimens scored as sound by the IOS was 52.2% with sound DIAGNOdent readings and 47.8% with carious DIAGNOdent readings. All samples identified as carious by the Trios4 were also identified as carious in the DIAGNOdent readings (Table 6).

**Table 6:** Concordance between Trios4 and diagnodent caries status

	Trios4 scoring		Kappa	p
	Sound	initial and moderate/extensive		
LF reading	n (%)	n (%)	0.454	**< 0.001**
Sound (< 30)	60 (52.2)	0 (0)		
Caries (≥ 30)	55 (47.8)	71 (100)		

Regarding the distribution of lesion scoring of Trios4, 61.8% of the lesions considered sound while 38.2% of the lesions considered caries. The rate of those with sound, initial, and moderate/extensive Trios4 scorings are 61.8%, 15.1%, and 23.1%, respectively (Table 7).

**Table 7:** Distribution of the Trios4 scoring

Trios4 scoring	Frequence	Percentage (%)
Sound	115	61.8
Caries (Initial or moderate/extensive)	71	38.2
IOS score		
Sound	115	61.8
Initial	28	15.1
Moderate/extensive	43	23.1

In the distributions of 3D Master scale matchings regarding the Trios4 scoring, most of the sound specimens corresponded to 2M3 shade (31.3%). Out of a total of 28 lesions scored as initial lesions, 20 of them matched the color 5M3. The Trios4 scored 43 of the 186 lesions as moderate/extensive, and 39 of them corresponded to 5M3 shade (Table 8).

**Table 8:** Distribution of vita 3D master scale according to Trios4 scoring

Vita 3D Master scale	IOS score		
	Sound	Initial	Moderate/Extensive
2L25	1 (0.9)	0 (0)	0 (0)
2M2	1 (0.9)	0 (0)	0 (0)
2M3	36 (31.3)	0 (0)	0 (0)
3L25	7 (6)	0 (0)	0 (0)
3M3	9 (7.8)	0 (0)	0 (0)
3R25	5 (4.3)	0 (0)	0 (0)
4M3	4 (3.5)	1 (3.6)	0 (0)
4R15	1 (0.9)	0 (0)	0 (0)
4R25	19 (16.5)	0 (0)	2 (4.7)
5M1	13 (11.3)	6 (21.4)	2 (4.7)
5M2	1 (0.9)	1 (3.6)	0 (0)
5M3	18 (15.7)	20 (71.4)	39 (90.6)

**Fig. 5:**
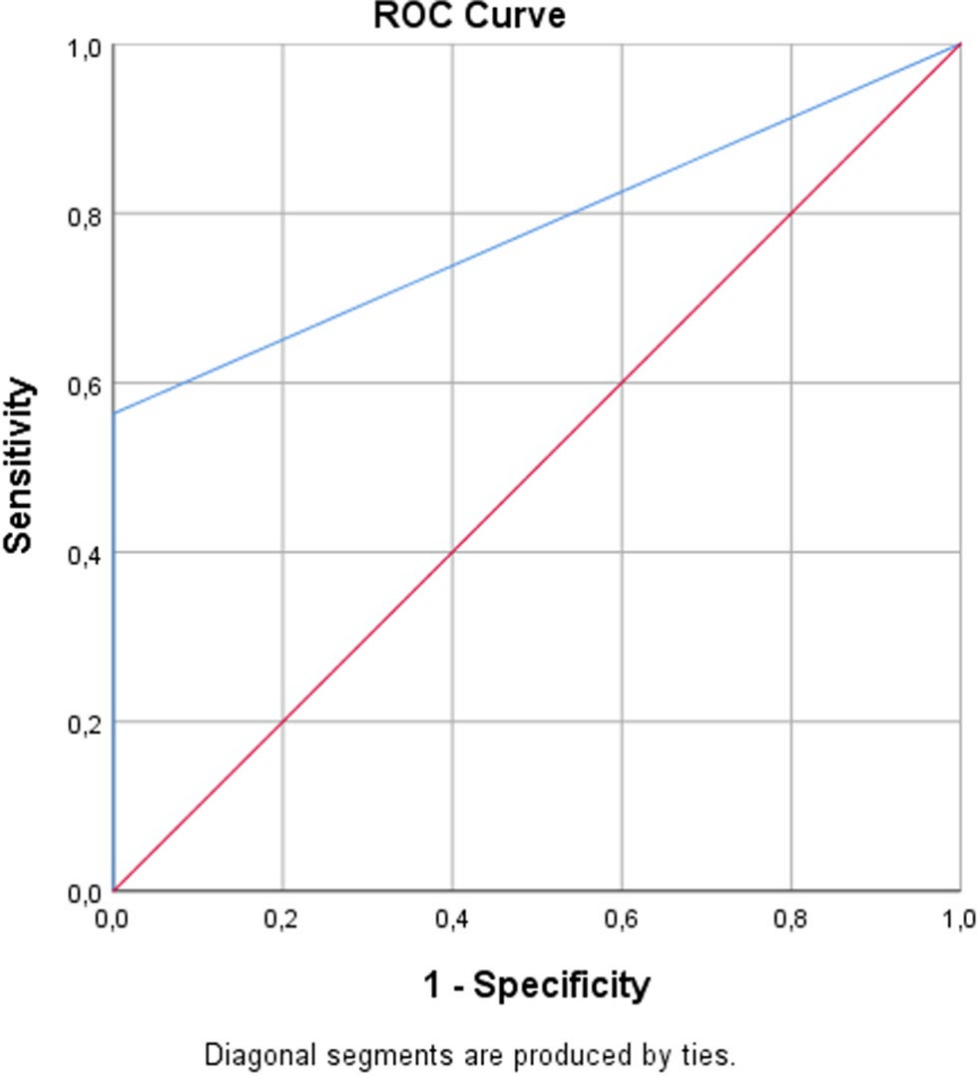
ROC curve of the Trios 4 according to the DIAGNOdent Pen

According the DIAGNOdent, Trios 4 scorings presented 0.782 AUC, 56.30% sensitivity, 100% specificity, 100%PPV, 52.17% NPV and 56.30% accuracy (Table 9. Receiver Operating Characteristic (ROC) curve of the Trios4 was given in Fig. 5.

**Table 9:** Sensitivity, specificity, and accuracy of the Trios 4 scoring according to diagnodent readings

	AUC (%95 CI)	*p*	Cut off	Sensitivity	Specificity	PPV	NPV	Accuracy
Trios 4 scoring	0.782 (0.718–0.845)	0.000	≤ 30	56.30%	100%	100%	52.17%	56.30%

## Discussion

The accuracy of the cavity-based assessment of the caries diagnostic method, which is included in the 3Shape software and advertised as fluorescence-based, is unknown. There is no guideline for the use of the caries aid software on the cavity floor, nor is there a recommendation to do so in the user manual. Thus, it should be noted that the present study focused on DIAGNOdent, which examines caries with a fluorescence-based assessment to understand the working principle and accuracy of the caries aid tool, unlike previous publications, and on comparison with data from a clinical-type contact spectrophotometer, which offers an advanced optical assessment [32, 33]. All measurements were performed in the dark box, taking into account the possibility of light interference with these sensitive optical systems [2, 34].

Colorimetric assessment occupies a considerable place in daily dental treatment practice. Therefore, clinicians needed numerical shade analysis to bring standardization to shade reproduction. This need has led to the development of spectrophotometric instruments. Subsequently, traditional shade guides like Vita Classic have been replaced by modern techniques. Moreover, white-balanced digital photographs using cross-polarized filters and grey reference cards with standardized set-up allow for accurate shade analysis in programs such as Adobe Photoshop [35–37]. Some commercial devices such as Spectroshade (MHT Optic Research, Italy) and Rayplicker and Rayplicker that collect images with a self-cross polarization filter and perform digital color assessment on them. Previous research compared the reliability of two spectrophotometer with a reference laboratory spectroradiometer. The Spectroshade device was found more reliable than Vita Easyshade (Vita Zahnfabrik, Bad Sackingen, Germany) device in colorimetric assessment [32]. Therefore, a clinical contact spectrophotometer with the same feature as the Spectroshade, but more up to date in the shade acquisition procedure, was chosen for our study.

Previous researchers have attempted to objectively evaluate the L*, a* and b* parameters of carious dentin stained with caries detection dye [38]. Researchers also used PCR for the detection of residual bacteria in remaining dentin. As the L* parameter increased, the CDD staining rate of the samples decreased. Furthermore, they stated that when the L* parameter was greater than 60, the remaining dentin was free of bacterial infection. They also observed that L* parameter was more significant than a* or b* parameters in detecting bacterial infection using caries detector dye. In the present study, a strong, negative correlation was found between the L* parameter and DIAGNOdent readings, a mechanism based on the fluorescence of bacterial metabolites, supporting the previous research’s findings [38].

Iwami et al. [6] evaluated the relationship between L*, a*, b* parameters of carious dentin and DIAGNOdent readings on eight extracted molars. Similar to the present results, they reported a significant negative correlation between L* parameter and DIAGNOdent readings (-0.853; *p* < 0.05). Conversely, the correlation for a* and b* parameters were not considered significant (*p* = 0.108 for a*,*p* = 0.018 for b*,). The large sample size in the present study may have resulted in carious dentin layers containing multiple stages of caries. This may have led to the variation in the red-green axis (a*) and yellow-magenta axis (b*) and the difference in fluorescence due to colour. In addition, in the previous study, a calculation was made using the Adobe Photoshop program with colour matching sticks. The difference in methodology between the two studies may have led to different results for a* and b* parameters.

In another study, a quantitative approach for determining the caries stage was investigated by correlating the spectral and chromatic features of caries lesions [2]. Samples with carious lesions were noted to have an additional fluorescence peak at approximately 627 nm. As the ICDAS score increased, the fluorescence intensity around 620 nm became more pronounced. This finding is supported by previous studies showing that red fluorescence around 600 nm is associated with bacterial porphyrins [39, 40]. They also reported a decrease in y with an increase in the x chromaticity coordinate (x: red; y: green color) as lesion severity increased [2]. In the present study, DIAGNOdent readings positively correlated with a* parameter, which is partially in agreement with the results of this study.

In another previous study, researchers compared two techniques to correlate spectral features of caries lesions with chromatic features, which were fast formula fitting and neural network fitting formulas [3]. These methods were applied to the quantitative classification estimation of dental caries and its visualization in a chromaticity diagram. Carious teeth were stimulated with a 405 nm LED light source, after which the spectral data of 334 test points were acquired by a hyperspectral imaging camera as the original dataset. Similar to Chen et al. [2], they observed a significant peak at 680 nm wavelength in the ICDAS 3,4 score. They also stated that both methods provide accurate estimation results at the pixel level [3]. They also concluded that 470–780 nm spectral power distribution was proved to be the best matching color waveband guiding the selection of filters in future instrument development [3]. Shortly after, Charvat et al. [5] introduced a novel method, diffuse reflectance spectroscopy, for caries detection. The diffuse reflectance spectroscopy (DRS) method showed superior performance to DIAGNOdent Pen. They attributed this result to the fact that diffuse reflectance spectroscopy uses a wide range of wavelength in the visible and near-infrared spectrum, compared to the narrow-spectrum laser devices used in caries diagnosis. The authors noted that DRS can show loss of light reflectance in the blue-green region of this broad spectrum (< 570 nm) [41], and increased reflectance at longer wavelengths (especially in 800–1000 nm) in carious teeth (increased water/decreased mineral content) [4]. Since this relationship is thought to affect the performance of optical caries diagnostic methods such as DIAGNOdent Pen, the relationship between dentin and color parameters has been investigated previously. Neves et al. [24] investigated the relation between L* parameter of remaining dentin and DIAGNOdent readings in in vitro conditions. They made image acquisition using a stereomicroscope illuminated by a 150 W light source-coupled to a digital camera. They obtained L*a*b* from RGB images using Image J software. Similarly to our study, they found a strong negative correlation between the L* parameter and DIAGNOdent readings. Based on the existing literature, it appears that parameters such as mineral density and water loss change the color, and indirectly the light reflection.

In the present study, a significant positive correlation was observed between DIAGNOdent readings and Trios4 scoring. Thus, the first hypothesis should be rejected. Although these results seem to indicate a predictable similarity between the two fluorescence-based caries diagnostic tools, it should be noted that the wavelengths of the devices are different (415 nm for Trios4, 655 nm for DIAGNOdent Pen). Fluorescence-based assessment of carious teeth relies on bacterial metabolites’ fluorescence under the illumination of red light [42]. This is the fundamental principle of the DIAGNOdent Pen for diagnosing carious lesions. However, some researchers have proposed an alternative hypothesis, suggesting that bacterial metabolites may not be the sole contributor to the increased DIAGNOdent Pen readings. Another perspective was that the autofluorescence of whiter teeth may be expected to be lower than that of discolored teeth [18]. Our findings appear to support this hypothesis, indicating that DIAGNOdent readings are higher in discolored surfaces. In addition, Trios 4 detected less caries than DIAGNOdent. It would seem that there is an increasing positive correlation with discoloration, which is less pronounced in Trios4. In light of these findings, it seems reasonable to conclude that Trios4 scoring at the 415-nm wavelength is less affected by the colorimetric parameters of the caries affected dentin than DIAGNOdent readings at the 655-nm wavelength.

The DIAGNOdent Pen has been used by many researchers at various cut-off values to determine the end point of caries removal. Alleman et al. [13] recommended DIAGNOdent Pen readings to obtain an intact peripheral dentin during caries removal. Yonemoto et al. [43] reported that outer caries dentin could be removed under the guidance of DIAGNOdent readings between 11 and 20 cut-off points. However, the study did not provide information on staining of dentin or lesion activity. Another study reported that the use of the DIAGNOdent Pen during caries removal at 39 cut-off point had a higher sensitivity, specificity, and accuracy than caries detection dyes [21]. Kanar et al. [44]. investigated the accuracy of DIAGNOdent Pen readings after caries removal. Overall, they found an accuracy of 86.49% for the DIAGNOdent Pen at a < 38 cut-off point. The authors also found that the residual caries risk after caries removal was 3.273 times higher in the ICDAS 4 score and 8.632 times higher in the ICDAS 5 score compared to the ICDAS 3 score. In light of this information, the peripheral seal zone concept can be recommended on deep dentin lesions using the DIAGNOdent Pen around the 30 cut-off point to provide both a minimally invasive endpoint and to ensure the sealing of the restoration. The first research regarding the fluorescence-based caries scoring system in the intraoral scanner was published by Michou et al. in 2020 [26]. They developed a caries scoring system using the fluorescence signals on the carious surfaces in vitro conditions. The intraoral scanner performed slightly better in terms of total sensitivity and specificity, and intra-examiner reliability compared to visual inspection. Subsequently, the accuracy of this caries scoring system, compared under in vivo and in vitro conditions, was conclusively reported with similar reliability [45].

Fluorescence-assisted caries excavation (FACE) is one of the proposed methods to determine the end point of caries removal. In an in vitro study, the bacterial diversity of soft dentin, leathery dentin and hard dentin was investigated using a fluorescence-based device which emits light at a wavelength of 405 nm (SIROInspect; Sirona, Bensheim, Germany). The researchers found that the diversity of bacteria was higher in the soft dentin than in the leathery and hard dentin. However, the degree of fluorescence detected by FACE did not correlate with the hardness of the remaining dentin [46]. In another study, the microhardness and shear bond strength of the remaining dentin after FACE and conventional caries removal were compared. Both methods did not presented a significant difference in terms of microhardness and shear bond strength of the remaining dentin [47]. Another in vitro study investigated the effect of heat generated by FACE and the remaining dentin thickness on the pulp of primary teeth. The authors reported that heat generation increased as the distance to the pulp chamber roof decreased, and the duration of FACE use increased. However, they did not find increased heat significant for causing irreversible pulpitis [48]. The authors agreed that further in vitro and in vivo studies needed to confirm these findings.

Sa et al. [49] investigated the reliability of a tooth-color-based ICDAS scoring on 3D digital images (by Trios 4) of 73 patients. Based on the ICDAS criteria, they graded the stages of caries as sound, initial, moderate and extensive, using fluorescence color descriptions ranging from green-yellow-orange to red on fluorescence model, and on tooth-colored 3D model. For all lesions, they reported an accuracy of 0.802 for assessment using tooth-colored 3D models and 0.810 for fluorescence-aided assessment. Similarly, Schulz- Weidner et al. [50] reported a 0.826 area under curve parameter for Trios 4 IOS, in enamel lesions, and 0.816 for dentinal caries lesions.

A review of the literature regarding the IOS shows that assessment on colored 3D models and fluorescence signals have considerable diagnostic power. Probably, in the near future, these 3D color images will form a diagnostic infrastructure for the creation of artificial intelligence networks. Indeed, the factors affecting imaging and hence diagnostic capacity, such as the amount and color of light, distance of the scanning head to the dentin surface, the color of the dentin, etc., are certainly significant of further investigation.

The present study focused on the spectrophotometric analysis of the remaining dentin and its fluorescence (DIAGNOdent readings) as one of these possible influencing parameters. Our findings showed that IOS scoring and L* parameter of the remaining dentin was inversely correlated. Moreover, IOS scoring significantly related with a* and b* parameters. Therefore, the second null hypothesis should be rejected. The possible relationship of a* and b* with DIAGNOdent readings is already discussed in this text. However, the lack of any previous study regarding the a* and b* parameters associated the Trios4 scoring in the literature limits the discussion. Yet, DIAGNOdent and Trios4 agreed on the soundness of 61 surfaces, and caries status of 71 surfaces (Table 6). However, the DIAGNOdent readings (for the 30 cut-off point) still indicated more remaining dentin caries than the Trios4. Regarding the Trios4 scoring, the mean of DIAGNOdent measurements for sound samples were 29, which is consistent with the cut-off point for dentinal caries lesions by DIAGNOdent, introduced by Lussi et al. [18]. However, one of the interesting findings is that the dataset indicates higher DIAGNOdent parameters for Trios4 caries detection. Recently, Michou et al. [51] assessed the agreement between IOS- integrated caries scoring system and conventional visual and radiographic data over thirty months. Following the re-examination of 55 participant after 30 months, the Trios4 detected significantly less caries progression. The authors noted that Trios4 tends to underestimate lesion severity and therefore recommended its use as a complementary method for caries diagnosis. Similarly, Jones et al. [52] stated that the IOS scoring system is weaker in identifying initial lesions and underestimates the severity of the lesion compared to ICDAS scoring. This research might be partially related to the findings of the present study in that it diagnoses the remaining dentin as less “carious” than DIAGNOdent readings. However, when caries removal is the primary concern, the authors of this article believe that these diagnostic methods should not lead to over preparation. Whether the lower caries detection at the endpoint of caries removal compared to DIAGNOdent readings should be considered a disadvantage or an advantage is a topic for debate. Moreover, future studies are required to understand the advantages and disadvantages of Trios4 scoring. This study addressed the color-related factors and DIAGNOdent readings of Trios4 scoring in the evaluation of remaining dentin. However, further large-scale studies with different fluorescence methods may expand the use of Trios4.

This study has some limitations. Although there is a defined subjective limit for the end-point of caries removal as ‘selective removal up to soft or firm dentin’, there is no consensus on the existence of a gold standard that will allow this to be measured quantitatively. Therefore, further clinical studies might be useful to evaluate the caries tissue presence for a more accurate and reliable outcome. Also, different fluorescence-based diagnostic methods can be compared in this regard. Moreover, this study did not evaluate the caries removal technique, and its influence on the diagnosis, while it might be an effective factor.

Within the limitations of this in vitro study, the following conclusions can be drawn;

- Trios4 scoring tends to predict remaining dentin as less carious than DIAGNOdent readings (for 30 cut-off point). This may reduce the tendency for over-preparation and can be considered as a contribution to minimally invasive dentistry.Both DIAGNOdent Pen and fluorescence-based caries scoring system of Trios4 correlate with the L* a* parameters of the remaining dentin.
- Studies comparing various fluorescence- based assessment methods under in vivo conditions could be carried out to confirm the results.

## Data Availability

The datasets used and/or analyzed during the current study are available from the corresponding author upon reasonable request.

## Declarations

### Concent to participate

Informed consent was obtained from each patient for using extracted teeth during the study.
